# Safety Profile of Nifurtimox and Treatment Interruption for Chronic Chagas Disease in Colombian Adults

**DOI:** 10.4269/ajtmh.15-0256

**Published:** 2015-12-09

**Authors:** Mario Javier Olivera, Zulma M. Cucunubá, Carlos Arturo Álvarez, Rubén Santiago Nicholls

**Affiliations:** Grupo de Parasitología, Instituto Nacional de Salud, Bogotá DC, Colombia; Red Chagas Colombia, Bogotá DC, Colombia; Departamento de Medicina Interna, Universidad Nacional de Colombia, Bogotá DC, Colombia; Dirección Científica, Clínica Universitaria Colombia, Colsanitas SA, Bogotá DC, Colombia

## Abstract

Nifurtimox (NFX) is one of the approved drugs used to treat Chagas disease. Safety profile studies and models on risk factors for treatment interruption in adults are scarce in Latin America. This study evaluated retrospectively the medical records of adult Chagas disease patients treated with NFX between 2007 and 2012 in Bogotá, Colombia. An accelerated failure time model was used, and associations were expressed as time ratio (TR). In total, 76 adult patients with NFX were included: 60 (79.0%) completed 60 days of treatment, 61 (80.3%) presented adverse drug reactions (ADRs), and 16 (21.0%) required treatment interruption. The predominant symptoms were epigastric pain (23.7%), nauseas (18.4%), sleep disturbances (18.4%), loss of appetite (17.1%), and temporary loss of memory (15.2%). ADRs were classified as mild (64.5%), moderate (30.4%), and severe (5.1%). Time of treatment was significantly longer when presenting ≤ 3 ADRs (TR: 1.78; 95% CI: 1.04–3.03), presence of non-severe ADRs (TR: 6.52; 95% CI: 3.24–13.1), doses of NFX ≤ 8 mg/kg/day (TR: 1.78; 95% CI: 0.90–3.49), and age < 48 years (TR: 1.57; 95% CI: 0.90–2.74). Treatment with NFX in adults caused a high frequency of ADRs, but most of the cases were mild and did not require treatment interruption. Severity and number of ADRs were the main predictors for treatment interruption.

## Introduction

Chagas disease is caused by the parasite *Trypanosoma cruzi* and still represents a serious public health problem in Latin American countries, where approximately 8–10 million people are infected.^1^,^2^ In Colombia, there is no certain information about the magnitude of the problem, but it is estimated that there are about 437,960 infected people, of which approximately 131,388 already present chronic cardiomyopathy.^3^ Without treatment, the infection progresses to the chronic phase. Symptoms appear usually decades after the initial infection,^4^ and about 30% of the infected patients progress to chronic chagasic cardiomyopathy.^5^ This is characterized by complex ventricular arrhythmias, bradyarrhythmias, atrioventricular block, apical aneurysm, ventricular dysfunction, and heart failure.^6^ Morbidity and mortality in this phase are mainly due to heart failure, sudden death, and pulmonary or cerebral embolisms.^7^

There are only two drugs available for treatment, benznidazole (BZN) and nifurtimox (NFX).^8^ They are recommended for both the acute and chronic phase, but their efficacy has been mainly proved in terms of reducing parasitemias and antibody titers.^9^ Treatment efficacy for stopping the progression of the heart disease is still in debate.^10^ The expected benefits of using these drugs include a reduction in the incidence of chronic complications and death.^9^,^11^ In adult population, treatment with NFX has been associated with about 80% probability of adverse drug reactions (ADRs)^12^,^13^ leading to interruption of treatment in up to 75% of patients.^14^ The most common adverse reactions reported have been gastrointestinal disturbances followed by neurological disorders.^12^,^15^ Children seem less susceptible to ADRs than adults, but these observations have not been clearly characterized.^15^–^18^

Most of the previous studies on adverse effects to treatment of Chagas disease have been based on univariate methods and mainly for BZN. For this reason, recent studies aimed to identify variables associated with the occurrence of ADRs using predictive models. By using survival analysis or time-to-event model, Jackson and others^12^ in Geneva, Switzerland, found that the presence of > 3 ADRs were associated to premature treatment termination with NFX. Given that there are not new drugs available for treating Chagas disease, it is important to have tools to identify patients with higher probabilities of developing ADRs and to design strategies to minimize complications and treatment interruption.

In Colombia, the first treatments for Chagas disease patients were with BZN in 2000, whereas NFX was introduced in the country in 2006, and then an important donation was received in 2008, which lead to generating the first experiences of treatment with NFX in children^19^ and adult populations. This study aimed to evaluate the tolerance and safety profile of NFX in a cohort of adult patients with chronic Chagas disease in Colombia and to identify the risk factors for treatment interruption.

## Methods

Observational and retrospective study of a cohort with follow-up during 60 days, conducted by reviewing medical records of patients with chronic Chagas disease who were treated with NFX at the National Health Institute of Colombia from June 2007 through December 2012.

### Patients and procedures.

Participants were included in the study if they fulfilled the following inclusion criteria: 1) ≥ 18 years old, 2) confirmed diagnosis of Chagas disease on the basis of two simultaneously positive IgG serological test results (enzyme-linked immunosorbent assay and indirect fluorescence antibody test) following international recommendations,^4^ 3) having received treatment with NFX, and 4) patients with completed clinical records for treatment.

Patients received treatment with NFX (Lampit^®^, Bayer HealthCare, El Salvador, Nicaragua) for 60 days, at a daily dose of 7–9 mg/kg divided into two or three times a day and clinical follow-up at the start, middle, and end of the treatment. Dose, duration of treatment, and follow-up regimes where conducted as recommended by the National Treatment Guidelines in Colombia^20^ and in other countries,^21^,^22^ and the drugs were donated by the Colombian Ministry of Health. Follow-up control included clinical and biological evaluations (complete blood count, liver and renal function tests, urinalyses, and serum creatinine levels) on days 0, 20, 40, and 60.

Patients had the possibility of daily consultation when needed and were instructed on managing ADRs before consultation. During each control, patients were briefed on the occurrence of expected ADRs. They also spontaneously reported other adverse effects that were then recorded. Specific ADRs were medically treated according to the National Treatment Guidelines in Colombia; the mild and moderate events were treated firstly with medications according to specific symptoms (analgesics for headache, proton pump inhibitors for epigastric pain, and anti-H2 for allergies). If this measure was not enough, then temporary drug suspension was implemented for a few days and then the drug was reintroduced at a lower dose that was gradually increased until reaching the prescribed dose.^20^ For these cases, the total treatment duration exceeded 60 days, but the duration of treatment with the full dose according to weight was always 60 days.

Causality of ADRs was assessed by using the Naranjo algorithm, a questionnaire of 10 questions to assess the causality of a variety of clinical situations of ADRs associated with a single drug. By using scores for each question ranging from −1 to +2, the event is assigned to a category of probability based on the total score. A total score of ≥ 9 is “definite,” 5–8 “probable,” 1–4 “possible,” and ≤ 0 “doubtful.”^23^ Severity of ADRs was classified into four categories: mild, moderate, severe, and fatal according to the level of affectation of the normal life of the patient.^24^

For the associations on the multivariate analysis, time to event was defined as the time in days from the start of treatment until definite discontinuation due to ADRs, and then called time to interruption.

### Statistical methods.

Results were presented with means, standard deviations, and medians. The Kolmogorov–Smirnov test was used to test the sample distribution. Continuous variables were analyzed with the Student's *t* test when normally distributed or with Mann–Whitney *U* test otherwise. Categorical variables were compared with Pearson's χ^2^ test or Fisher's exact test, as appropriate.

One researcher judged all ADRs using the Naranjo algorithm. The same researcher evaluated all ADRs 2–3 weeks later, again using the Naranjo criteria. Test–retest reliability was assessed using the kappa statistic. The kappa coefficient was interpreted according to the guidelines proposed by Landis and Koch.^25^

Time-to-event curves (traditionally called survival curves) were constructed for time to interruption. Patients were censored at time of completion of treatment if no definite interruption had appeared. The analyses were restricted from 0 to 60 days of follow-up. For the relation of severity and interruption, a bivariate analysis was performed by the Kaplan–Meier method and compared by using the log-rank test. In addition, the worst case scenario was analyzed by including patients lost to follow-up.

Given that a semi-parametric proportional hazards assumption was not suitable to the data (proportional hazards implies a constant hazard for any of the covariates over time), a regression analysis with a Cox proportional hazards model was considered not feasible.^26^ For that reason, we assessed five different parametric distributions from the general family distribution (exponential, Weibull, log-logistic, log-normal, and generalized gamma). To determine the most appropriate distribution, the lowest Akaike Information Criterion (AIC) value was considered as the best fit.^27^ Once the best distribution was chosen, a log-logistic accelerated failure time-to-event model with gamma frailty was obtained. This allows an expectation–maximization algorithm to estimate the parameters, and it is the most common distribution used in the frailty model.^28^,^29^

Because of the fact that time ratios (TRs) are more amenable with parametric models, they were used for measuring the association between exposure and survival experience (time to interruption). For TR(*p*), the time required for *p* percent of individuals in the exposed population (to covariates) to experience the event of interest (definite interruption) is TR(*p*)-fold the time for the same proportion of events to occur in the reference population.^30^ Then, TR is defined as “relative quantiles of survival time,” for 0 < *P* < 1 as the ratio of the corresponding quantile functions, which is as follows^30^:

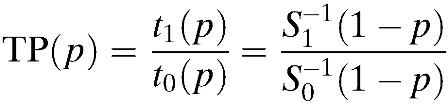


The general interpretation of TR(*p*) is that a TR > 1 implies that the variable is associated with a longer time from the start of NFX until stopped due to ADRs, whereas a TR < 1 the other way around.^29^

Statistical analyses were performed using the Stata^®^ (release 11.0) software package (Stata Corp., College Station, TX) and R statistical package (R Development Core Team, 2008, Vienna, Austria)^31^. *P* values less than 0.05 were considered as statistically significant.

### Ethical approval.

Approvals were granted by the Technical Research Committee and Ethics Research Board at the National Health Institute in Bogotá, Colombia: protocol CTIN-014-11, Minute 9 of December 11, 2012. Participation was voluntary and patients were asked for informed written consent to access information on their clinical records. To ensure confidentiality, the information was accessed through a coded system.

## Results

### Univariate analysis.

Between 2007 and 2012, 85 records of patients treated with NFX were identified. Of these, one patient did not have the complete clinical record and eight were lost to follow-up treatment. In total, 76 patients were included in this study. The mean age was 42.1 years (standard deviation: 11.5, range: 18–72), and 51 (67.1%) were women. The characteristics of patients lost during the follow-up did not differ from those who continued monitoring.

### Frequency of ADRs.

From 76 patients treated with NFX 60 (79.0%) completed 60 days of treatment and 16 (21.0%) required definite treatment interruption because of intolerance to NFX. The exact dose of NFX ranged from 360 to 780 mg/day (mean: 539.5 ± 88.4) and the duration of treatment ranged from 9 to 72 days (mean: 53.6 ± 13.2). As shown in Table 1, of the 76 patients treated, 61 (80.3%) patients had at least one ADR during the use of NFX and 53 (86.9%) of them had > 3 ADRs; only one patient (1.6%) had one ADR. All ADRs disappeared after discontinuation of treatment.

Specific reported symptoms in order of frequency were epigastric pain in 18 (23.7%), nausea in 14 (18.4%), abdominal bloating in 13 (17.1%), sleep disturbances in 14 (18.4%), temporary loss of memory in 12 (15.8%), headache in 12 (15.8%), loss of appetite in 13 (17.1%), myalgia in 11 (14.5%), and eosinophilia in 9 (11.8%) of the cases, as shown in Table 2. The frequency of incidental ADRs of any severity was not different during the range of days evaluated at each 20 days monitoring: 15.3% (95% confidence interval [CI]: 8.40–24.73) from 0 to 20 days, 11.1% (95% CI: 5.20–20.04) from 20 to 40 days, and 11.8% (95% CI: 5.56–21.29) from 40 to 60 days.

The symptoms that lead to definitive interruption of treatment in order of frequency were epigastric pain in three (3.9%), headache in two (2.6%), abdominal bloating in one (1.3%), vomiting in one (1.3%), urticaria in one (1.3%), skin peeling in one (1.3%), paresthesia in one (1.3%), depression in one (1.3%), sleep disturbances in one (1.3%), loss of appetite in one (1.3%), myalgia in one (1.3%), lymphocytosis in one (1.3%), and increased AST levels in one (1.3%) of the cases.

### Causality and severity of ADRs.

In this study, overall 217 ADRs were identified (see Table 2). Regarding the causality assessment using the Naranjo algorithm, 25 (11.5%) ADRs were classified as definite, 80 (36.9%) as probable, 51 (23.5%) as possible, and 61 (28.1%) as doubtful. The intra-observer kappa for assigning causality to NFX was κ = 0.85 (95% CI: 0.81–0.91). In terms of severity, 140 (64.5%) cases were classified as mild, 66 (30.4%) as moderate, and 11 as severe (5.1%). The intra-observer kappa for assigning severity to NFX was κ = 0.93 (95% CI: 0.91–0.97).

### Bivariate analysis for ADRs.

**Figure 1.**
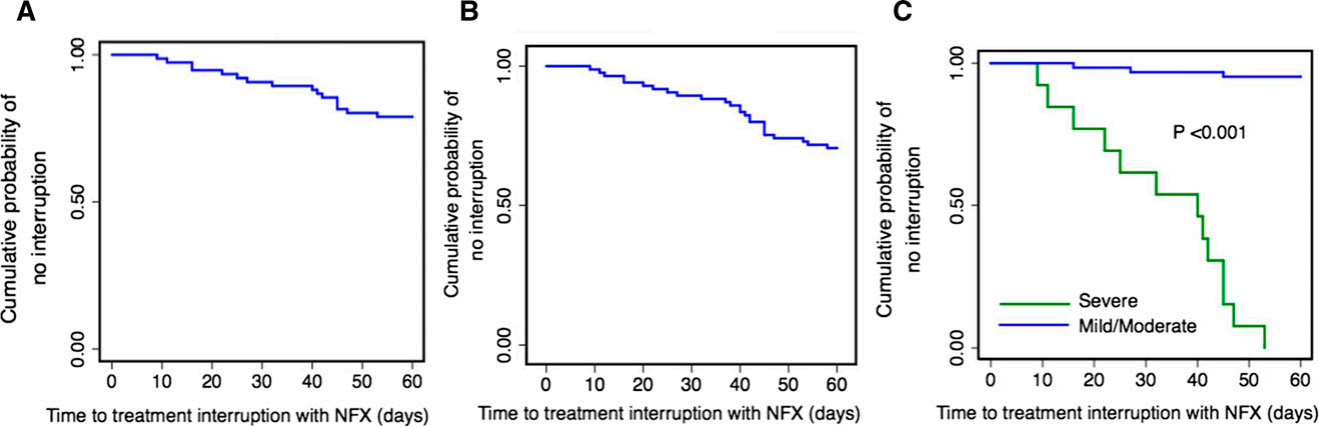
Kaplan–Meier curves for free time of treatment interruption with nifurtimox (**A**) general, (**B**) including the patients lost to follow-up (worst case scenario), and (**C**) by severity of adverse drug reactions (ADRs).

The average time to interruption was 32.3 days (95% CI: 24.6–39.8). The Kaplan–Meier curves showed that frequency of treatment interruption did not vary across the study period (Figure 1

). This analysis stratified by the severity of ADRs indicates that the median time to interruption was shorter in patients with severe ADRs compared with patients with mild and moderate ADRs. Variables with a *P* value < 0.1 in the bivariate analysis were considered for inclusion in the multivariate model. In addition, by clinical experience and literature review, we decided to include age as a covariate in the multivariate analysis, see Table 3.

### Multivariate analysis for ADRs.

**Figure 2.**
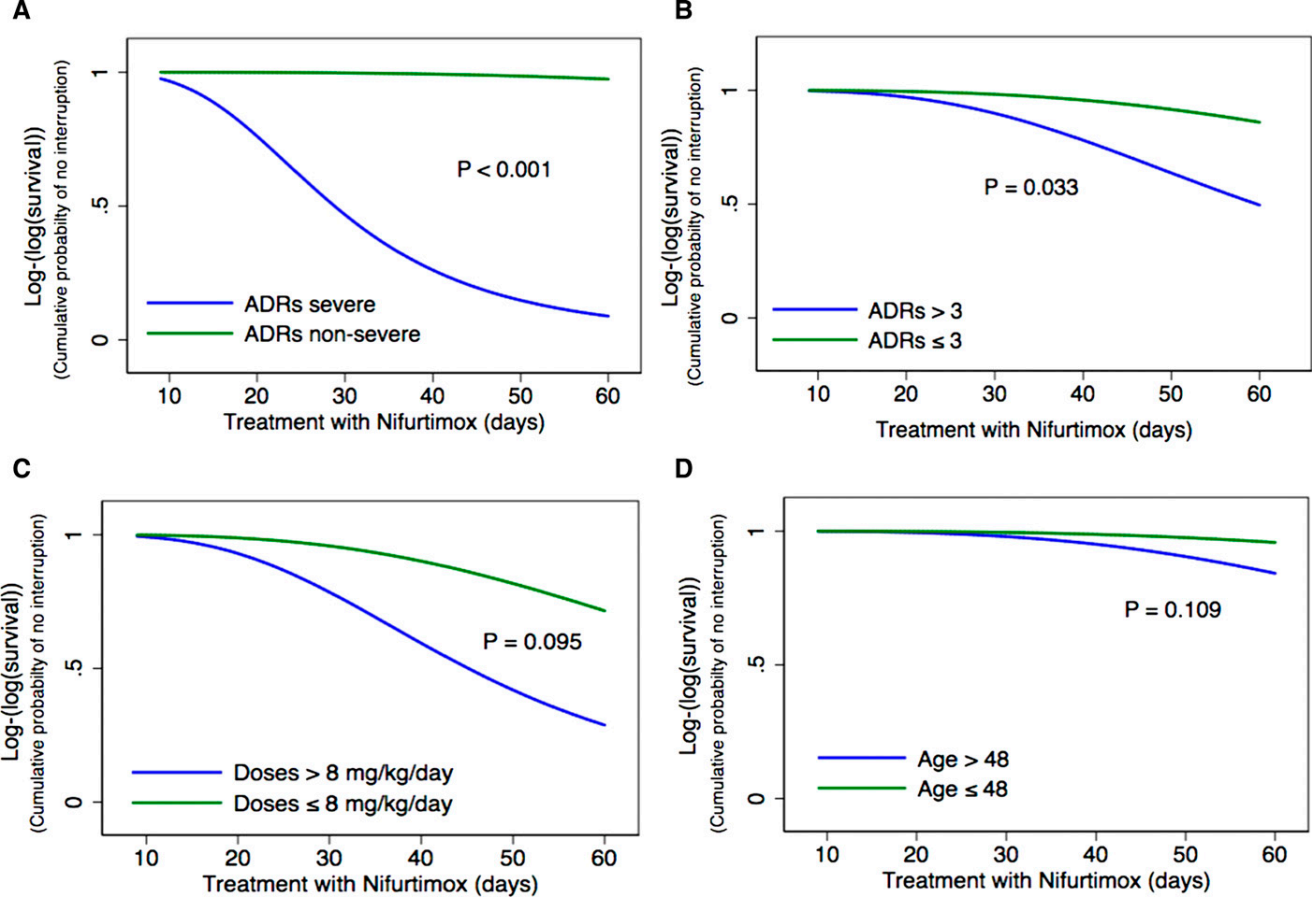
Accelerated failure time model with log-logistic distribution and gamma frailty for risk factors associated with time free to treatment interruption: (**A**) severity of adverse drug reactions (ADRs), (**B**) numbers of ADRs, (**C**) dose, and (**D**) age.

The results of the comparison between parametric models show that log-logistic distribution was the best candidate according to the AIC value (54.4) compared with the others: exponential (68.1), Weibull (59.2), log-normal (64.1), and generalized gamma (60.9). According to the final multivariate model, time of treatment was longer for age < 48 years (TR: 1.57; 95% CI: 0.90–2.74), presence of ≤ 3 ADRs (TR: 1.78; 95% CI: 1.04–3.03), dose of NFX ≤ 8 mg/kg/day (TR: 1.78; 95% CI: 0.90–3.49), and absence of severe ADRs (TR: 6.52; 95% CI: 3.24–13.1) (Table 3). Survival curves showing the time to interruption for each risk factor of the final model are shown in Figure 2

.

## Discussion

This study shows that definitive interruption of treatment with NFX in adults infected with *T. cruzi* is significantly associated with the severity and the number of ADRs, followed by dose and age. Even though the ADRs were common (80.3%), only in 21.0% of cases required definitive interruption. Out of these 76 patients who received NFX, 80.3% had at least one ADR. A similar incidence of ADRs, 75%, was reported in a previous prospective cohort study conducted in 1997 in Brazil^14^ and Spain.^15^ Other prospective studies have reported higher frequencies, from 88.1% in Chile^13^ up to 97.5% in adult Latin American immigrants in Switzerland.^12^ However, other studies have reported lower frequencies of ADRs, 29.6–32.0%.^17^,^32^

Coinciding with previous findings in adult patients treated with NFX, this study found that gastrointestinal symptoms and neurological disturbances were predominant.^12^,^13^,^15^ Most of the patients (60/76) had more than one ADR, and noncumulative changes such as peripheral neuropathy and bone marrow depression were observed,^12^,^32^ which can be perceived at any time during treatment.^13^

In this study, 79.0% of patients completed the full 60-day course of treatment, and in 21.0% patients, definitive interruption was needed. These results were in contrast with previous studies that have reported a higher percentage of interruption. In Brazil, the disruption of treatment has been reported at between 29.6% to 75% and of patients,^14^,^32^ whereas in Argentina these reports have been lower between 3.7% and 23%.^17^,^33^ However, in those studies the time to interruption of treatment was not mentioned. In our study, the average time to interruption was 32.3 days (95% CI: 24.6–39.8), which contrasts with previous reports of 9 days (interquartile range [IQR]: 8–10),^15^ and 14 days (95% CI: 10.2–17.8).^12^

In the context of survival analysis, traditionally the most frequently used method is the proportional hazard regression (Cox) model, for which the interpretation of the parameters is directly linked to relative hazards.^30^ However, we were not only interested in the relative hazards but in quantifying how exposures modify the magnitude of time to interruption. A major advantage of the accelerated failure time-to-event model on the standard Cox model is that it measures the effects of covariates (acceleration/deceleration) over survival time, provides complete description of the data, and the results are easily interpreted, allowing a clear distinction between the effects of covariates on the timing and the limiting survival probability of the event.^26^ With respect to the survival distribution, it was expected that a log-logistic distribution fit to the data best than any other parametric survival model because it gives a non-monotonic hazard function that increases at early times and decreases at later times, which is sensible to the clinical occurrence of ADRs to NFX.^27^

In our study, ADRs' severity and the presence of > 3 ADRs were the best predictors for premature interruption of treatment. These finding confirm previous results of a study using a Cox proportional hazard model with NFX, where the presence of > 3 ADRs determined a hazard ratio of 8.4 (95% CI: 1.6–45.5) and was the most important predictor for premature interruption.^12^ An association of premature interruption with the severity of ADRs had been previously described for BZN.^34^

It is important to highlight that most of the factors associated with treatment interruption are not modifiable, except from the doses that was slightly less than 8 mg/kg/day in some cases. NFX was administered at the usual dose and for the recommended duration in ambulatory care (60 days) in Colombia, and the treatment average was 52.9 days. Although currently there is no evidence of the consequences of decreasing doses on the efficacy of the treatment, clearly this is one of the topics that future research can be focused, assessing the efficacy of either lower doses or shorter duration of the etiological treatment aimed at reducing the occurrence of ADRs.

Even though age was not found as a clear statistically significant risk factor for the duration of the treatment, this finding could be explained either by the small sample size involved in this study or by the absence of such association. Nevertheless, in the bivariate analysis it is evident that the frequency of ADRs is higher at older ages, and previous studies have suggested that the probability of ADRs increases with age, being 1.5 times higher in patients over 65 years of age (odds ratio [OR]: 1.5; 95% CI: 1.08–2.09).^35^ This effect has been explained by pharmacokinetic, pharmacodynamic, and homeostatic changes combined with the effect of coexisting diseases and the increase of polypharmacy with age.^36^ Interestingly, a recent published study that evaluates treatment of children with NFX found that only 4.8% of cases required temporary suspension to achieve 100% adherence to the 60-day treatment.^19^

Although a high frequency of ADRs was observed in women, female sex was not found as a predictor of NFX interruption due to ADRs. In a recent study using BZN, it was observed that female sex predicted ADRs that caused interruption of treatment (OR: 2.3; 95% CI: 1.2–4.3).^37^

Assessing causality between the use of a drug and the development of ADRs is a complex process, because this relationship is usually caused by the joint action of a main factor (drug) and other cofactors that facilitate their appearance (risk factors).^38^ Although, there is not a perfect method for assessing causality, by using the Naranjo algorithm, our study presented an almost perfect agreement in assigning causality of ADRs.

Although several follow-up studies of adverse effects of trypanocidal drugs have been published in recent years, most of them have been based on BZN.^33^,^39^ In addition, the study of NFX is highly important because alternative treatments different to BZN and NFX have not yet proved a similar efficacy and also because in small series it has been found that NFX can be used safely in patients who discontinued previous treatment with BZN due to any adverse reaction.^15^

The limitations of this study are inherent of facility-based data and the retrospective approach. The existence of NFX has fluctuated in the international market,^40^ and for this reason the number of patients treated with this drug in Colombia is still low. Nevertheless, clinical records had a specific section for the reporting of expected and unexpected ADRs that were systematically recorded, and this study represents the experience of the largest cohort of adults receiving NFX in the country.

In conclusion, even though the frequency of ADRs during administration of NFX was high (80.3%), most of the ADRs were considered mild and only 21.0% of cases required definite treatment interruption. Because of the high frequency of ADRs, clinical follow-up is recommended during the treatment course. The accelerated failure time-to-event model was useful as a predictive model for time to interruption based on the clinical characteristics of each patient. Several factors associated with less probability of treatment interruption were identified. These results can be used to guide the search of strategies to improve tolerance to NFX and for patient stratification criteria for randomization in clinical trials.

## Figures and Tables

**Table 1:** Proportion of ADRs to NFX stratified by variables of interest

Variable	Category	ADRs	
		Yes	No
		*n* (%)	*n* (%)
Sex	Female	40 (78.4)	11 (21.6)
	Male	21 (84.0)	4 (16.0)
Age	18–48 years	39 (79.6)	10 (20.4)
	> 48 years	22 (81.5)	5 (18.5)
Interruption of treatment	Yes	16 (100)	0 (0)
	No	45 (75.0)	15 (25.0)
Total		61 (80.3)	15 (19.7)

ADRs = adverse drug reactions; NFX = nifurtimox.

**Table 2:** Frequency of ADRs to NFX stratified by systems and symptoms

Systems or organs	Frequency		Symptom	Frequency	
	*n*	% (*n*/217)		*n*	% (*n*/76)
Gastrointestinal	67	30.9	Epigastric pain	18	23.7
			Nausea	14	18.4
			Abdominal bloating	13	17.1
			Abdominal pain	6	7.9
			Vomiting	6	7.9
			Diarrhea	4	5.3
			Constipation	3	3.9
			Dysphagia	3	3.9
Central and peripheral nervous system	46	21.2	Sleep disturbance	14	18.4
			Temporary loss of memory	12	15.8
			Lack of concentration	9	11.8
			Depression	6	7.9
			Vertigo	3	3.9
			Paresthesia	2	2.6
Body as a whole, general disorders	24	11.1	Headache	12	15.8
			Adynamia	6	7.9
			Asthenia	5	6.6
			Fever	1	1.3
Autonomic nervous system	18	8.3	Loss of appetite	13	17.1
			Increase in appetite	5	6.6
Musculoskeletal	17	7.8	Myalgia	11	14.5
			Arthralgia	6	7.9
Skin and appendages	17	7.8	Exanthema	6	7.9
			Itching	5	6.6
			Urticaria	3	3.9
			Bullous eruption	2	2.6
			Skin peeling	1	1.3
Hematological	17	7.8	Eosinophilia	9	11.8
			Leukopenia	3	3.9
			Neutrophilia	3	3.9
			Lymphocytosis	2	2.6
Liver	11	5.1	Increased AST levels	6	7.9
			Increased ALT levels	5	6.6
Total	217				

ADRs = adverse drug reactions; ALT = alanine aminotransferase; AST = aspartate aminotransferase; NFX = nifurtimox.

**Table 3:** Bivariate and multivariate analysis by AFT model for time to interruption (of NFX treatment)

Covariate	Bivariate analysis				Multivariate analysis			
	Coefficient (βi)	TR exp(βi)	95% CI	*P* value	Coefficient (βi)	TR exp(βi)	95% CI	*P* value
Severity								
Severe	Ref	Ref	–	–	Ref	Ref	–	–
Mild/moderate	1.82	6.20	(2.84–13.6)	**< 0.001**	1.88	6.52	(3.24–13.1)	**< 0.001**
Doses mg/kg/day								
> 8	Ref	Ref	–	–	Ref	Ref	–	–
≤ 8	1.01	2.77	(0.89–8.59)	0.077	0.57	1.78	(0.90–3.49)	0.095
Age (years)								
≥ 48	Ref	Ref	–	–	Ref	Ref	–	–
< 48	−0.08	0.92	(0.34–2.49)	0.874	0.45	1.57	(0.90–2.74)	0.109
ADRs								
> 3	Ref	Ref	–	–	Ref	Ref	–	–
≤ 3	0.92	2.51	(1.39–4.51)	**0.002**	0.58	1.78	(1.04–3.03)	**0.033**
Sex								
Male	Ref	Ref	–	–	–	–	–	–
Female	0.55	1.72	(0.74–4.01)	0.203	–	–	–	–
History of previous pathologies								
Yes	Ref	Ref	–	–	–	–	–	–
No	0.67	1.95	(0.82–4.64)	0.129	–	–	–	–
Alteration of leukocytes								
Yes	Ref	Ref	–	–	–	–	–	–
No	0.01	1.01	(0.23–4.40)	0.921	–	–	–	–
Alteration of lymphocyte								
Yes	Ref	Ref	–	–	–	–	–	–
No	0.29	1.33	(0.70–2.56)	0.378	–	–	–	–
Alteration of neutrophils								
Yes	Ref	Ref	–	–	–	–	–	–
No	0.46	1.59	(0.51–5.01)	0.426	–	–	–	–
Alteration of eosinophil								
Yes	Ref	Ref	–	–	–	–	–	–
No	0.08	1.08	(0.57–2.07)	0.795	–	–	–	–

ADRs = adverse drug reactions; AFT = accelerated failure time model; CI = confidence interval; NFX = nifurtimox; Ref = reference variable; TR = time ratio.

Bold values are statistically significant results *P* < 0.005.
